# Study Protocol PROMETHEUS: Prospective Multicenter Study to Evaluate the Correlation Between Safety Margin and Local Recurrence After Thermal Ablation Using Image Co-registration in Patients with Hepatocellular Carcinoma

**DOI:** 10.1007/s00270-022-03075-5

**Published:** 2022-03-01

**Authors:** Timo T. M. Oosterveer, Gonnie C. M. van Erp, Pim Hendriks, Alexander Broersen, Christiaan G. Overduin, Carla S. P. van Rijswijk, Arian R. van Erkel, Rutger W. van der Meer, Maarten E. Tushuizen, Adriaan Moelker, Martijn R. Meijerink, Otto M. van Delden, Koert P. de Jong, Christiaan van der Leij, Maarten L. J. Smits, Thijs A. J. Urlings, Jeffrey P. B. M. Braak, Elma Meershoek-Klein Kranenbarg, Bianca van Duijn-de Vreugd, Evelijn Zeijdner, Jelle J. Goeman, Jurgen J. Fütterer, Minneke J. Coenraad, Jouke Dijkstra, Mark C. Burgmans

**Affiliations:** 1 Department of Radiology, Leiden University Medical Center, PO Box 9600, 2300 RC Leiden, The Netherlands; 2 Division of Image Processing, Leiden University Medical Center, Leiden, The Netherlands; 3 Department of Medical Imaging, Radboud University Medical Center, Nijmegen, The Netherlands; 4 Department of Gastroenterology and Hepatology, Leiden University Medical Center, Leiden, The Netherlands; 5 Department of Radiology and Nuclear Medicine, Erasmus University Medical Center, Rotterdam, The Netherlands; 6 Department of Radiology, Amsterdam University Medical Center, Amsterdam, The Netherlands; 7 Department of Hepatobiliary and Pancreatic Surgery, University Medical Center Groningen, Groningen, The Netherlands; 8 Department of Radiology, Maastricht University Medical Center, Maastricht, The Netherlands; 9 Department of Radiology, University Medical Center Utrecht, Utrecht, The Netherlands; 10 Department of Radiology, Haaglanden Medical Center, Hague, The Netherlands; 11 Department of Surgery, Leiden University Medical Center, Leiden, The Netherlands; 12 Dutch Oncology Research Platform, Utrecht, The Netherlands; 13 Department of Biomedical Data Sciences, Leiden University Medical Center, Leiden, The Netherlands

**Keywords:** Hepatocellular carcinoma, Thermal ablation, Minimal ablation margin, Local recurrence, Co-registration

## Abstract

**Purpose:**

The primary objective is to determine the minimal ablation margin required to achieve a local recurrence rate of < 10% in patients with hepatocellular carcinoma undergoing thermal ablation. Secondary objectives are to analyze the correlation between ablation margins and local recurrence and to assess efficacy.

**Materials and Methods:**

This study is a prospective, multicenter, non-experimental, non-comparative, open-label study. Patients > 18 years with Barcelona Clinic Liver Cancer stage 0/A hepatocellular carcinoma (or B with a maximum of two lesions < 5 cm each) are eligible. Patients will undergo dual-phase contrast-enhanced computed tomography directly *before* and *after* ablation. Ablation margins will be quantitatively assessed using co-registration software, blinding assessors (i.e. two experienced radiologists) for outcome. Presence and location of recurrence are evaluated independently on follow-up scans by two other experienced radiologists, blinded for the quantitative margin analysis. A sample size of 189 tumors (~ 145 patients) is required to show with 80% power that the risk of local recurrence is confidently below 10%. A two-sided binomial *z*-test will be used to test the null hypothesis that the local recurrence rate is ≥ 10% for patients with a minimal ablation margin ≥ 2 mm. Logistic regression will be used to find the relationship between minimal ablation margins and local recurrence. Kaplan–Meier estimates are used to assess local and overall recurrence, disease-free and overall survival.

**Discussion:**

It is expected that this study will result in a clear understanding of the correlation between ablation margins and local recurrence. Using co-registration software in future patients undergoing ablation for hepatocellular carcinoma may improve intraprocedural evaluation of technical success.

*Trial registration* The Netherlands Trial Register (NL9713), https://www.trialregister.nl/trial/9713.

## Introduction

Hepatocellular carcinoma (HCC) is the third most common cause of cancer-related death in the world [1]. Surgical resection is the preferred treatment for patients with HCC ≥ 2 cm, but resection may not be feasible or perilous as a result of unfavorable tumor location or underlying liver disease [1]. Thermal ablation is an alternative treatment with lower complication rates, lower costs, and shorter hospital stay [2]. However, hepatic resection yields better results regarding local recurrence (LR) [2].

To reduce the risk of LR after thermal ablation, it is generally recommended to ablate a tumor with a minimal ablation margin (MAM) of > 5 mm [3]. A clear relation between MAM and LR seems evident, but the precise relationship still needs to be established. Also, there is no validated, standardized method to accurately determine a MAM. Commonly, margins are assessed through side-by-side positioning of pre-and post-ablation cross-sectional images and visual *qualitative* assessment. Over recent years, co-registration software has become available that allows immediate three-dimensional *quantitative* assessment of the MAM. It would potentially be the equivalent of the frozen section that is used for margin control during surgery. Yet, quantitative margin assessment during ablation has not been validated in large prospective studies and is not common practice.

Quantitative margin assessment can only determine intra-procedural treatment decisions if the correlation between MAM and LR is clearly understood. In this study, obtained margins will be *quantitatively* assessed and correlated with clinical outcomes. The primary objective is to determine the MAM required to achieve a local recurrence rate (LRR) of < 10% in patients with HCC [4]. Secondary objectives are to analyze the correlation between MAM and LR and to assess efficacy of thermal ablation in patients with HCC.

## Materials and Methods

### Trial Design and Study Setting

The PROMETHEUS trial is a prospective, multicenter, non-experimental, non-comparative, open-label study. The sponsor of the study is the Leiden University Medical Center (LUMC). This study is a collaboration between Dutch academic centers and cancer organizations. The trial is funded by the Dutch Cancer Society and registered at https://www.trialregister.nl (ID: NL9713).

### Participants

Patients over 18 years with Barcelona Clinic Liver Cancer stage 0-A HCC, or stage B with a maximum of two lesions < 5 cm each, are eligible. Full inclusion and exclusion criteria are provided in Table 1.

**Table 1:** Full inclusion and exclusion criteria

Inclusion criteria	Exclusion criteria
Age 18 years or above HCC very early (0) or early-stage (A) according to the BCLC staging system, OR HCC intermediate stage (B) according to the BCLC staging system with a maximum of two lesions of ≤ 5 cm each Either de novo or recurrent HCC: prior locoregional therapy is allowed in the study^a^ Candidate for percutaneous thermal ablation as discussed in a multidisciplinary tumor board Absence of any psychological, familial, sociological, or geographical condition potentially hampering compliance with the study protocol and follow-up schedule Written informed consent	Estimated GFR < 30 ml/min Known severe allergy to contrast medium ASA classification > 3 Child-Pugh C Tumor related ECOG ≥ 1 Neoadjuvant transarterial therapy (TACE, TAE, or TARE), i.e. combination therapy of transarterial therapy Portal vein tumor invasion Extrahepatic metastasis Uncorrectable coagulopathy

Abbreviations: *ASA* American Society of Anesthesiologists, *BCLC* Barcelona Clinic Liver Cancer, *ECOG* Eastern Cooperative Oncology Group, *GFR* glomerular filtration rate, *HCC* hepatocellular carcinoma, *TACE* transarterial chemoembolization, *TAE* trombo-endarterectomy, *TARE* transarterial radioembolization

^a^Recurrence in an area with prior TACE or TARE treatment is considered to be combination therapy and thus excluded. In case of prior TACE/TARE treatment, only recurrence in another area of the liver may be included

### Interventions

**Fig. 1:**
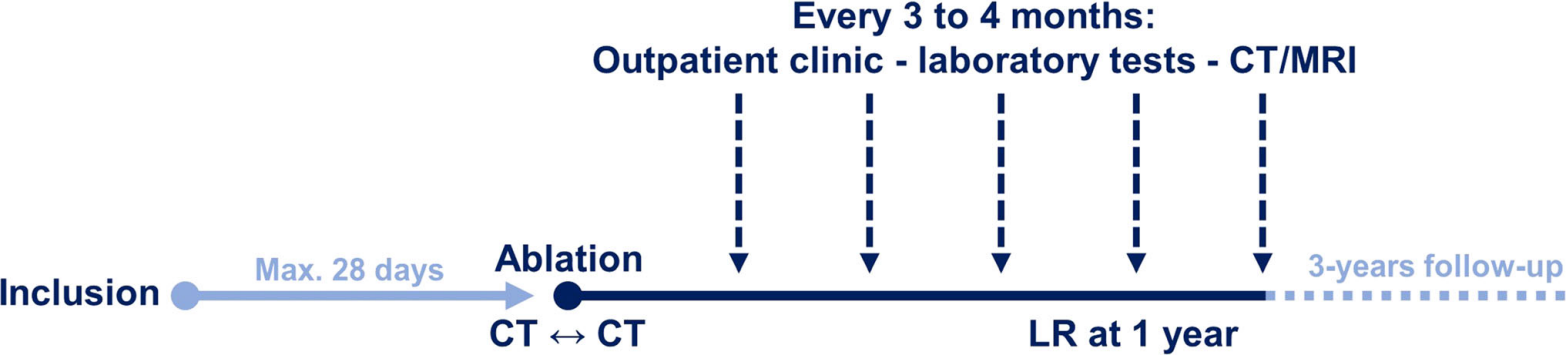
Schematic overview of interventions and major time points for participants

All patients are discussed in a multidisciplinary tumor board for eligibility and consented prior to inclusion. The ablation procedure and follow-up will be according to local standard of care. Interventions and important time points are shown in Fig. 1.

Both radiofrequency ablation (RFA) and microwave ablation (MWA) are allowed in the study. All patients will undergo dual-phase contrast-enhanced computed tomography (CECT), i.e. arterial and venous phase, directly *before* and *after* the ablation. Ablation and CECT will be performed under general anesthesia. The pre-ablation and post-ablation CECT will be performed during apnea to minimize breathing artifacts. Alternatively, high-frequency jet ventilation may be used.

At the end of the procedure, the interventional radiologist will determine whether complete tumor ablation with sufficient margins was achieved. All patients will be treated with the intent to obtain complete tumor ablation with a > 5 mm margin and it is left at the discretion of the treating interventional radiologist to determine whether technical success has been achieved. Assessment will be performed as per current practice, i.e. visual qualitative assessment in most centers. Peri-procedural care will be in accordance with the protocol of the local institution.

### Follow-Up

Patients will undergo physical examination, laboratory tests, contrast-enhanced magnetic resonance imaging of the liver and chest-CT every 3–4 months after ablation until liver transplantation, untreatable progression, or death. Follow-up scans will be reviewed independently by two experienced interventional radiologists, other than the radiologists assessing the quantitative MAM (see MAM analysis), to determine the presence and location of recurrence. These radiologists will be blinded for the analyses of the quantitative MAM. Disagreement between the two radiologists will be resolved by consensus reading.

### MAM Analysis

**Fig. 2.**
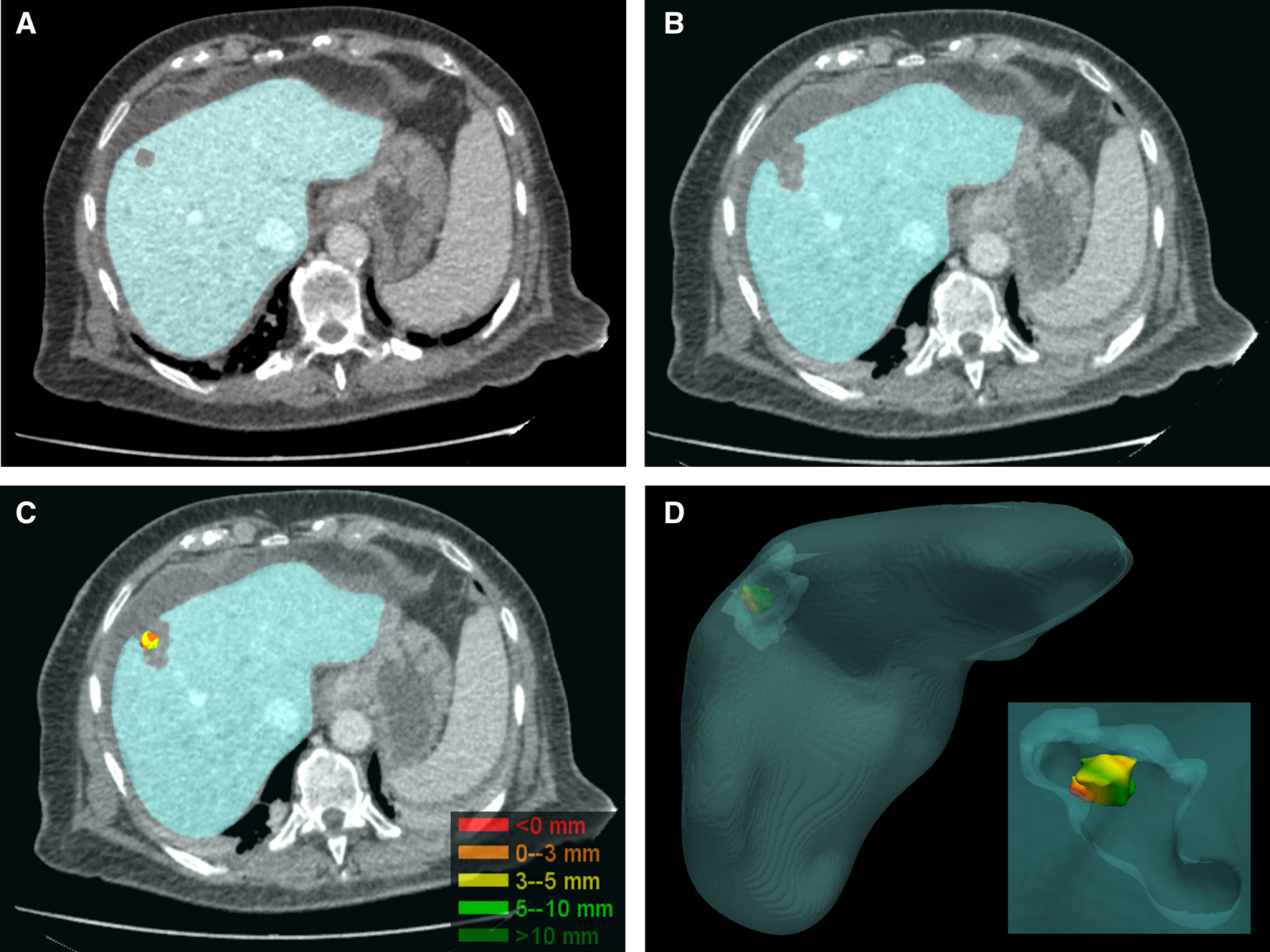
3D Quantitative MAM assessment using deLIVERed in a 78 years old female with a single HCC of 1 cm. **A** Axial slice showing the liver mask based on delineation of the liver and tumor on the pre-ablation venous phase CT-images. **B** Axial slice showing the liver mask based on delineation of the liver and ablation zone in the venous phase post-ablation CT-images. **C** 2D representation of the 3D tumor model with color-coded ablation margins after co-registration. Ablation margins are calculated in 3D, hence showing unexpected tight medial ablation margins which are not visible in 2D. **D** 3D model of the tumor and ablation zone with color-coded ablation margins after co-registration

All pre- and post-ablation CECT images will be transferred online to the LUMC using ALEA Clinical (FormsVision, The Netherlands). Two experienced interventional radiologists, blinded for outcome, will independently perform delineation of the tumor and ablation zone, on the pre-and post-ablation CECT respectively. The pre-and post-ablation CECT will be co-registered using post-processing software (deLIVERed, LUMC) to quantitatively assess the MAM (Fig. 2). Discordances of > 3 mm between both radiologists will be resolved by consensus reading, otherwise, the mean MAM will be calculated. The mean MAM will then be correlated with the presence and location of LR. The results of deLIVERed will be compared with SAFIR (Fraunhofer-Gesellschaft) software to determine whether results are reproducible using different co-registration software. All clinical data will be entered in Castor Electronic Data Capture and subsequently analyzed using appropriate software packages (SPSS or R).

### Outcomes

#### Primary Endpoint

The primary endpoint is the MAM that results in an LRR < 10% at 1-year follow-up.

#### Secondary Endpoints

LR at 1 year will be analyzed for different MAM categories: < 0 mm, 0–3 mm, 3–5 mm, and $\ge$ 5 mm. Also, local and overall recurrence rates and disease-free survival (DFS) and overall survival (OS) at 1, 2, and 3 years will be analyzed. Finally, the relation between LR and DFS and OS will be investigated.

### Sample Size

A sample size of 189 tumors would be sufficient to show with 80% power that the risk of LR is confidently below 10%, assuming a true risk of 4% for tumors ablated with a MAM ≥ 2 mm, based on a study by Kim et al. [5]. Other retrospective studies into local recurrence provide similar numbers as provided by Kim et al. [5–7]. The calculation is based on the normal approximation of the binomial distribution (*d* = 0.10–0.04 = 0.06; *s* = sqrt(0.04 * 0.96) = 0.20; *z *= 1.96 + 0.84 (80% power); *N *= (s*z/*d*)^2^ = 84 tumors with ablation margin ≥ 2 mm; total number of tumors *N* = 84/0.445 = 189). Based on our own data, patients will have an average of 1.3 tumors per patient. Thus for 80% power, a sample size of 145 patients is indicated. Taking a potential drop-out rate of 10% into account, the needed sample size is 165 patients.

### Statistical Methods

For the primary objective, a two-sided binomial *z*-test will be used to test (reject) the null hypothesis that the LRR is ≥ 10% for patients with a MAM ≥ 2 mm. Additionally, as a secondary analysis, a logistic regression model will be used to find the relationship between MAM and LR. Kaplan–Meier estimates will be used to assess local and overall recurrence, DFS, and OS. Survival data will be censored at the date of last follow-up if patients are still alive. The log-rank test will be used to compare recurrence for different MAM categories. Logistic regression analyses will be performed to determine possible independent predictors for local and overall recurrence, DFS, and OS. A *p*-value < 0.05 will be considered significant.

## Discussion

Thermal ablation is on its way to replace surgical resection as the treatment of first choice for patients with early-stage HCC. Thermal ablation offers clear advantages over surgery in an era with rising healthcare costs and an aging population. The task that lies ahead is to bring the efficacy of thermal ablation up to par with surgical resection. Various studies have demonstrated that LRRs comparable to resection can be achieved if sufficient ablation margins are obtained.

Recommendations to ablate a liver tumor with a MAM > 5 mm are mainly based on expert opinion and pertain to treatment intent rather than the actual obtained margins. Ablation systems have predefined algorithms, based on in vitro experiments, to predict the size and shape of the ablation, but tissue factors influence the actual ablation volume and size. Several studies have demonstrated that true margins are often narrower than intended and often misjudged by conventional side-by-side evaluation of pre-and post-ablation images [5, 6, 8–11].

Retrospective studies have demonstrated the potential of quantitative MAM assessment using image co-registration. In a study including 110 patients with 176 HCCs, the MAM was assessed using CECT-CECT co-registration and proved to be the only significant independent predictor of local tumor progression (LTP) [6]. For each millimeter increase of the MAM, a 30% reduction of the relative risk for LTP was found (OR = 0.7, 95%CI 0.5–0.98, *p* = 0.036). No LTP was detected in lesions with a MAM > 5 mm, but only in 37.5% of tumors, this MAM was obtained. Similar results were reported in a study by Kim et al., which included 103 patients with 110 HCCs [5]. MAM was also assessed with CECT-CECT co-registration and strongly correlated with LRRs: 22.7%, 18.9%, 5.9%, and 0% for margins of ≥ 0 mm, ≥ 1 mm, ≥ 2 mm, and ≥ 3 mm, respectively. Remarkably, in only 2.7% of the ablations, the MAM was > 5 mm. Park et al. found that the cumulative incidence of LR was twice as high in patients with a MAM < 2 mm, compared with a MAM ≥ 2 mm [12]. Another retrospective study by Jiang et al. found similar results, but the post-ablation CT used for co-registration was obtained 1 month after ablation, and shrinkage of the ablation zone within this period may have led to underestimation of margins [7].

As PROMETHEUS is a prospective study with a standardized imaging protocol, it is expected that this study will result in a clearer understanding of the correlation between MAM and LR and in validation of quantitative margin analysis. Knowledge provided will be important for the implementation of image co-registration as an intraprocedural decision-making tool in clinical practice. In future patients, it may help to objectively identify areas at risk of LR and instigate re-ablation during the same treatment session if margins are deemed to be insufficient. Following the above-mentioned retrospective studies, PROMETHEUS is the next step towards clinical use of image co-registration as an intraprocedural decision-making tool.

Our study has several limitations. The study is designed as a prospective, single-arm observational study without control group. However, this is also a strength, as the PROMETHEUS study allows optimal and standardized imaging of the tumor and ablation zone during the same session. In addition, it might be that the optimal ablation margin is dependent on ablation size and type of ablation system used. It is allowed to include patients with intermediate-stage HCC with a maximum of two HCCs < 5 cm. However, it is common practice in most participating centers to treat patients with HCC > 3 cm with combined transarterial chemoembolization and ablation. These patients are not eligible for inclusion and we thus expect that the vast majority of patients will have tumors < 3 cm. In posthoc analysis, we will investigate whether differences in optimal MAM exist between patients treated with various ablation systems.

Furthermore, tissue contraction may pose an important challenge when interpreting our study results. Tissue contraction during ablation may result in calculated margins being smaller than they actually are. Currently, there is insufficient knowledge on how contraction is influenced by factors such as cirrhosis, tumor cellularity, ablation systems, power settings, and ablation times. Most studies on tissue contraction have been performed in healthy ex-vivo animal livers. Brace et al. studied the difference in contraction for MWA compared to RFA based on multiple markers in sections of healthy unperfused ex-vivo bovine livers [13]. The mid and peripheral markers, placed at a distance of 10 and 15 mm from the ablation applicator respectively, showed a significant difference in contraction between RFA and MWA. This difference was not seen for the inner markers, placed at 5 mm from the ablation applicator. Two in-vivo animal model studies report a tissue contraction up to 12% [14, 15]. However, this was also in normal liver tissue. One retrospective in-vivo human study was performed by Lee et al. [16]. In contrast to the study by Brace et al., they found a limited relative tumor and ablation zone contraction of −9.95% and −7.1%, respectively, for tumors treated with MWA [16]. The exact amount of tissue contraction in patients with HCC treated with thermal ablation remains unknown, may vary between patients and depends on liver consistency. However, as tissue contraction is present in all patients, it is indirectly taken into account in the cut-off value for the MAM.

Last, it is assumed that LRs for different tumors in the same patient are independent [4].

## Abbreviations

CECT: Contrast-enhanced computed tomography
DFS: Disease-free survival
HCC: Hepatocellular carcinoma
LR: Local recurrence
LRR: Local recurrence rate
LTP: Local tumor progression
LUMC: Leiden University Medical Center
MAM: Minimal ablation margin
MWA: Microwave ablation
OR: Odds ratio
OS: Overall survival
RFA: Radiofrequency ablation
